# Exploring the Pathological Journey of Superficial Spreading Squamous Cell Carcinoma of the Cervix Into the Endometrium

**DOI:** 10.7759/cureus.63788

**Published:** 2024-07-03

**Authors:** Ramya Chitturi, Dhatri D Muttavarapu, Radhika Medidi, Jyothsna Bingi, Aparna Chinnam

**Affiliations:** 1 Pathology, Guntur Medical College, Guntur, IND

**Keywords:** cervical cancer, cancer cervix, vagina, superficial spread, endometrium, cervical squamous cell carcinoma

## Abstract

The superficial extension of cervical squamous cell carcinoma (SCC) into the endometrium by replacing the endometrial glands is rare, as it normally spreads by invading the stroma or by lymphatic invasion. We present a case of a postmenopausal female complaining of vaginal discharge followed by vaginal bleeding. Microscopy showed a superficial spreading SCC of the cervix extending superficially into the endometrium with focal myometrial invasion. Carcinoma in situ changes were observed in the vagina. Based on the Fluhmann criteria and ancillary immunohistochemical testing, it was concluded to be an extension of cervical SCC and not primary endometrial carcinoma. The importance of this entity has not been given because of its low incidence.

## Introduction

Cervical carcinomas usually spread in the path of least resistance, either by directly invading the stroma or by lymphatic invasion of the uterine wall [1]. Superficial spread to the contiguous endometrium by replacing normal endometrial glands is rare. This entity has not been given due recognition in the latest World Health Organization (WHO) classification of female genital tract tumors [2]. The current American Joint Committee on Cancer's eighth edition of tumor staging also does not give relevance to the involvement of endometrium, vagina, or ovaries by cervical squamous cell carcinoma (SCC) [3]. The prognostic factors and optimal management have not been elucidated, as the number of cases reported in the literature is less than 100.

## Case presentation

**Figure 1:**
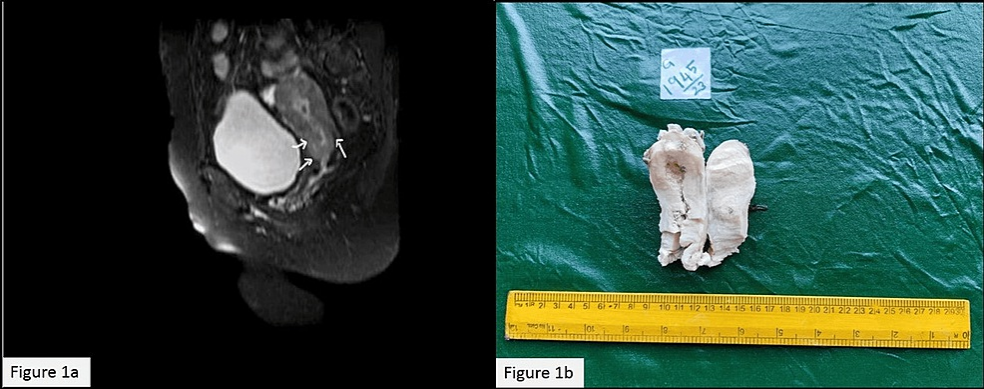
(a) MRI T2 weighted image: axial view of an ill-defined lesion in the endocervical canal extending superficially into the endometrium. (b) Gross appearance of the tumor extending from the cervix into the endometrium MRI: magnetic resonance imaging

A 46-year-old female who attained menopause two years ago came with the chief complaint of vaginal bleeding. Her history revealed that she had complained of white discharge for one week, followed by vaginal bleeding. Initially, the patient was advised to undergo a biopsy for further evaluation at a local hospital, but she opted for ayurvedic treatment, which provided temporary relief for two to three weeks. However, her symptoms recurred and persisted for an additional two months before seeking consultation at the gynecology department of the current hospital. The per vaginal and per speculum examinations did not reveal any significant findings. A cervical biopsy was performed, revealing a diagnosis of a high-grade squamous intraepithelial lesion (HSIL). MRI of the pelvis showed an ill-defined T1 hypo and T2 hyperintense lesion measuring 21 x 16 mm in the endocervical canal, extending superiorly to the lower one-third of the body of the uterus, involving the anterior and posterior myometrium, with loss of normal junctional zones (Figure 1a). A total abdominal hysterectomy with bilateral salpingo-oophorectomy and bilateral pelvic lymph node dissection was done.

The gross examination of the uterus revealed a gray-white growth measuring 6.5 x 2 x 1.5 cm, extending from the cervix to the body and fundus of the uterus (Figure 1b). Additionally, the inner surface of the vaginal cuff shows multiple gray-white granular areas in continuation with that of the cervix. Seven left-sided pelvic lymph nodes and two right-sided pelvic lymph nodes were isolated.

**Figure 2:**
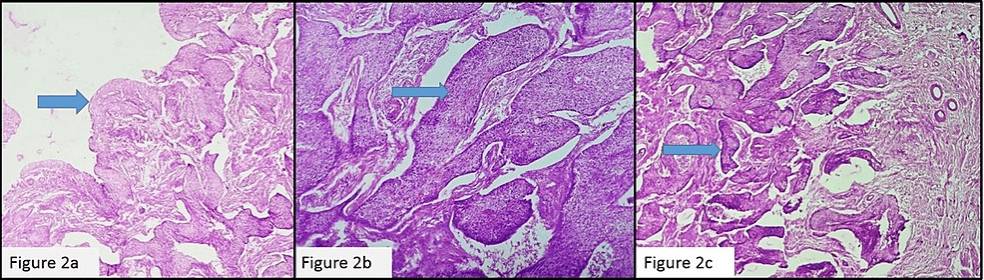
(a) HSIL 40x, (b) SCC cervix H&E 100x, and (c) tumor cells in the endometrium and invading the myometrium H&E 40x HSIL: high-grade squamous intraepithelial lesion, SCC: squamous cell carcinoma, H&E: hematoxylin and eosin

Microscopic examination of the cervix revealed an HSIL (Figure 2a) along with a moderately differentiated SCC with stromal invasion of 8 mm depth (Figure 2b). The tumor cells were extending superiorly into the endometrium, displacing the normal endometrial glands and invading the myometrium (Figure 2c).

**Figure 3:**
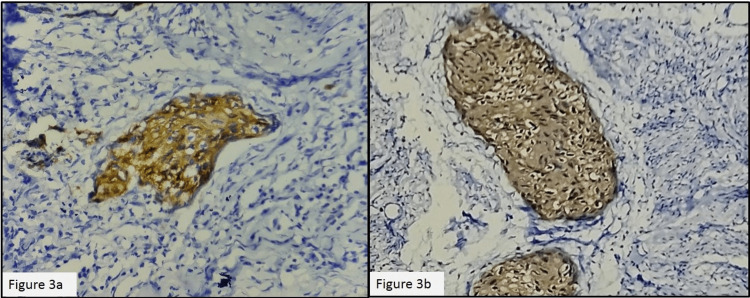
(a) p16 IHC in the cervix 400x and (b) p16 IHC in the endometrium 400x IHC: immunohistochemistry

Inferiorly, the tumor cells were extending into the vagina, with the epithelium revealing carcinoma in situ changes. Four out of seven left-sided pelvic lymph nodes and one out of two right-sided pelvic lymph nodes showed metastatic tumor deposits. Bilateral parametria were free from tumors. Both the ovaries and tubes were free of tumors. Pathological staging was pT2a N1a M0. Immunohistochemistry (IHC) with the p16 antibody was performed on sections from the cervix and endometrium and showed diffuse cytoplasmic positivity in tumor cells at both sites (Figure 3a-3b).

The patient was followed for up to six months without any significant comorbidities. No further chemotherapy or radiotherapy was given as the patient refused to undertake further treatment.

## Discussion

Cervical stenosis was found to be one of the risk factors for superficial spreading SCC of the cervix into the endometrium in many studies [4,5], but in the present case, we could not find any stenosis. There are two possible theories for the superficial spread of cervical carcinoma. One theory states the transformation of endometrial cells to squamous cells, whereas the other theory proposes contiguous/transtubal spread [6]. When genetic analysis of five cases of cervical SCC with synchronous superficial SCC of the upper genital tract was analyzed by Kushima et al., most of these carcinomas were found to be monoclonal neoplasms that were originating from the cervical mucosa and superficial migration of the tumor clone to the upper genital mucosa [7].

It is common in postmenopausal women, and the commonest presentation is vaginal bleeding, which was seen in the present case [8]. The intrauterine spread of cervical carcinoma in the endometrium can be identified as whitish patches on gross inspection, which is known as "cake icing" or Zuckerguss carcinoma [9]. This superficial spread can be missed grossly sometimes; hence, meticulous gross examination and adequate bits should be given in cervical carcinoma cases.

The superficial extension of cervical SCC should be distinguished from primary endometrial SCC. Fluhmann has established the following criteria for the diagnosis of primary endometrial SCC, which were later modified by Kay et al. [10,11]. They are the following: (1) no evidence of coexisting endometrial adenocarcinoma or primary cervical SCC, (2) no connection between the endometrial tumor and squamous epithelium of the cervix, and (3) no connection between any existing cervical in situ carcinoma and independent endometrial neoplasm.

Carcinoma in situ and microinvasive SCC were also reported to extend into the endometrium by superficial spread [12,13]. The tumor cells from the cervix can also extend into the fallopian tubes and ovaries, as reported by Gungor et al. and Agashe et al. [6,9].

Though most cases in the literature did not present with lymph node metastasis, our case presented with lymph node metastasis, which was similar to the case report by Kanbour et al. [14]. In node-negative cases, further follow-up studies have to be done to know whether a simple hysterectomy alone is sufficient rather than a radical surgery.

IHC with p16, Ki67, p63, and CD138 has been tried in many studies, but p16 was consistently positive in tumor cells from both the endometrium and cervix, reinforcing the theory of contiguous spread from the cervix into the endometrium [8]. p16 positivity in endometrial and cervical carcinoma also indicates the role of HPV in the carcinogenesis of superficial spreading cervical SCC. Even though some studies found that superficial spreading cervical SCC with endometrial involvement has a worse prognosis than primary endometrial SCC, this has to be confirmed by further investigations [14].

## Conclusions

A preoperative endometrial evaluation should be done along with a cervix biopsy in suspected cases of cervical carcinoma, especially in postmenopausal women. Although the recent WHO classification has not yet incorporated this variant, clinicians and pathologists must consider its existence. Further research is warranted to identify relevant prognostic factors and establish guidelines for optimal management.
